# Abilities of Pre-Treatment Inflammation Ratios as Classification or Prediction Models for Patients with Colorectal Cancer

**DOI:** 10.3390/diagnostics11030566

**Published:** 2021-03-21

**Authors:** Andra Ciocan, Răzvan A. Ciocan, Nadim Al Hajjar, Claudia D. Gherman, Sorana D. Bolboacă

**Affiliations:** 1 Department of Medical Informatics and Biostatistics, “Iuliu Hațieganu” University of Medicine and Pharmacy Cluj-Napoca, Louis Pasteur Street, No. 6, 400349 Cluj-Napoca, Romania; Andra.Ciocan@umfcluj.ro; 2 Department of Surgery, “Iuliu Hațieganu” University of Medicine and Pharmacy Cluj-Napoca, Croitorilor Street, No. 19-21, 400162 Cluj-Napoca, Romania; nadim.alhajjar@umfcluj.ro; 3 “Prof. Dr. Octavian Fodor” Regional Institute of Gastroenterology and Hepatology Cluj-Napoca, Croitorilor Street, No. 19-21, 400162 Cluj-Napoca, Romania; 4 Department of Medical Skills—Human Sciences, “Iuliu Hațieganu” University of Medicine and Pharmacy Cluj-Napoca, Marinescu Street, No. 23, 400337 Cluj-Napoca, Romania; gherman.claudia@umfcluj.ro

**Keywords:** inflammation biomarkers, colorectal cancer, derived ratios, prognostic factors

## Abstract

Background: Systemic inflammatory status is known as an important factor of colorectal cancer prognosis. Our study aimed to evaluate the performances of inflammation biomarker ratios as classification models of seven outcomes in patients with colorectal cancer. Methods: A retrospective cohort study was conducted on subjects with colorectal cancer over five years at a single center in Transylvania, Romania. Seven derived ratios were calculated based on laboratory data: neutrophil-to-lymphocyte (NLR), derived neutrophil-to-lymphocyte (dNLR), platelet-to-lymphocyte (PLR), lymphocyte-to-monocyte (LMR) and albumin-to-globulin (AGR) ratios, Systemic Immune Inflammation Index (SII) and Prognostic Nutritional Index (PNI). The utility of these ratios as predictors for seven outcomes was further evaluated in multivariable regression models. Results: Our study shows that the evaluated ratios exhibit specific performances for individual outcomes, proving a fair ability as screening tools (NLR and dNLR for survival, T stage and M stage; NLR and SII for T stage; and PLR for M stage). A dNLR over 3.1 (OR = 2.48, 95% CI (1.421 to 4.331)) shows predictive value for survival. A value of NLR over 3.10 (OR = 1.389, 95% CI (1.061 to 1.817)) is positively associated with an advanced T stage, while LMR is negatively related to the T stage (OR = 0.919, 95% CI (0.867 to 0.975)). NLR over 4.25 (OR = 2.647, 95% CI (2.128 to 3.360)) is positively associated with, while PNI is negatively related (OR = 0.970, 95% CI (0.947 to 0.993)) to, the M stage. Conclusion: Each of the evaluated ratios possesses prognostic value for certain outcomes considered, but the reported models need external validation to recommend their clinical practice utilization.

## 1. Introduction

Colorectal cancer is the third most common cancer in the world. Improvements in surgical techniques and management of patients with colorectal cancer have been seen, but the 5-year survival remains the same for those with metastatic disease [1]. Identification of biomarkers that can correctly predict disease status in the early stages is needed, thus reducing the cases of patients present in the health care system at the metastatic stage [2].

The incidence and mortality of colorectal cancer have doubled in the last 20 years in Romania, reaching an incidence of 17.74/100,000 inhabitants. In 2010, 8240 new cases were registered, placing Romania among the countries with an average disease incidence [3,4]. In 2018 and 2019, colorectal cancer was the second leading cause of cancer death (after bronchopulmonary cancer, but ahead of gastric cancer), with 4860 deaths per year [5,6].

The men to women ratio is 1.3, and less than 3% of cases occur in people under 40 years of age. The incidence increases rapidly over 45 years and doubles with each decade of life. About 2/3 of people diagnosed with bowel cancer are over 60 years old [7,8].

Early-stage colorectal cancer may be asymptomatic. The symptoms of advanced colorectal cancer are nonspecific and can also occur in people with benign diseases (such as hemorrhoids or colonic polyps). Early colorectal cancer symptoms include blood in the stools or rectal bleeding, change in the normal bowel habit persisting for more than three weeks, such as diarrhea, constipation or alternation constipation–diarrhea, abdominal pain, unexplained weight loss or paraneoplastic syndromes [9,10].

Surgical resection with total mesorectal excision represents the fundamental curative treatment for patients with rectal cancer. Neoadjuvant treatment protocols (concomitant chemotherapy and radiation) followed by radical surgery with/without adjuvant chemotherapy have led to decreased recurrence, improved surgical outcome and increased 5-year overall and disease-free survival. An accurate assessment of the tumor stage by imaging methods (such as computed tomography, magnetic resonance imaging or positron emission tomography) and biopsy is difficult preoperatively. In this sense, an attempt was made to determine biological markers that can better indicate these patients’ prognosis. Thus, various ratios (lymph node ration, Glasgow Prognostic Score, Peterson index, Klintrup score) have been studied on patients with colorectal cancer to determine patients’ status [11,12]. Systemic inflammatory status has an important impact on the prognosis of cancer in general [13]. Several biomarkers can be obtained before starting treatment and can be considered as prognostic factors, predicting the disease’s evolution [14,15]. Some biomarkers such as hemoglobin, neutrophil-to-lymphocyte ratio and platelet count should be able to predict tumor status and provide information about the tumor before surgical resection [16]. Prognostic inflammatory factors could include neutrophil-to-lymphocyte (NLR), platelet-to-lymphocyte (PLR), lymphocyte-to-monocyte (LMR) and albumin-to-globulin (AGR) ratios [17]. An increased NLR has been reported to be associated with a poor prognosis for colorectal cancer [18,19]. Further, an increase in the PLR ratio is associated with a lower survival rate [20] or is reported as having a limited value in colorectal cancer [17]. Similar results occur when evaluating the other ratios of systemic inflammation [21]. Consequently, further study of inflammation biomarkers is needed to optimize their diagnostic and prognostic values [22].

Our study’s primary objective was to evaluate seven ratios’ abilities as classification tools for seven outcomes in patients with colorectal cancer. The cutoff values identified in the primary objectives were used to construct multivariable models, in order to predict a specific outcome.

## 2. Materials and Methods

### 2.1. Study Design

A retrospective cohort study was conducted at the Third Surgical Clinic, “Prof. Dr. Octavian Fodor” Regional Institute of Gastroenterology and Hepatology Cluj-Napoca, Romania. Subjects admitted with the diagnosis of colorectal cancer between January 2014 and September 2019 were eligible for the study.

The medical records of the patients hospitalized during the study period were reviewed. The main criterion for inclusion in the study was given by the principal diagnosis of colonic or rectal cancer. Demographics (e.g., gender, age, environment), clinical data (e.g., complications, neoadjuvant chemo- or radiotherapy), laboratory tests (e.g., hemoglobin, total proteins, leucocytes, lymphocytes, neutrophils, thrombocytes, albumin, globulin, monocytes) and imaging staging (T stage—primary tumor, and M stage—presence of distant metastasis) were retrieved for the eligible patients. Patients with incomplete medical records (e.g., had missing data related to the pathology or did not undergo surgical treatment during hospitalization) were excluded from the study.

### 2.2. Outcomes and Derived Predictors

We analyzed seven outcomes: preoperative imaging stages T and M, neoadjuvant therapy (chemotherapy/radiation), feeding and transit resumption (expressed in days), presence of complications and death. The T stage was considered as follows: 1—tumor has grown into the submucosa, the layer of tissue underneath the mucosa; T2—tumor has grown into the muscularis propria; T3—tumor has grown through the muscularis propria and into the subserosa or it has grown into tissues surrounding the colon or rectum; T4—tumor has grown into or has attached to other organs or structures. The M stage was expressed as presence (M1) or absence (M0) of metastasis. The T and M stages were based on MRI (magnetic resonance imaging), helical CT (computer tomography) of 3 mm slices, contrast-enhanced ultrasonography or FDG (fluorodeoxyglucose)-PET (positron emission tomography), depending on the case.

**Figure 1:**
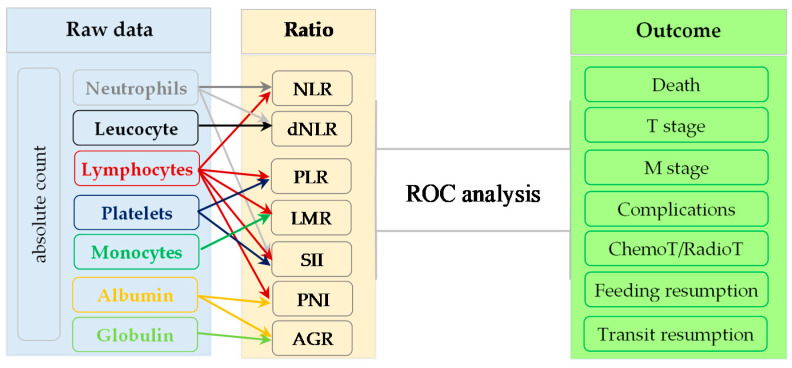
Input data for receiver operating characteristic (ROC) analysis. NLR = neutrophil-to-lymphocyte ratio; dNLR = derived neutrophil-to-lymphocyte ratio; PLR = platelet-to-lymphocyte ratio; LMR = lymphocyte-to-monocyte ratio; AGR = albumin-to-globulin ratio; SII = Systemic Immune Inflammation Index; PNI = Prognostic Nutritional Index. The raw data of three outcomes, namely, T stage, feeding resumption and transit resumption, were used to construct derived dichotomial variables as follows: T1 or T2 vs. T3 or T4 for T stage, resumption of feeding in the first 3 days post-intervention and resumption of transit in the first 4 days post-intervention.

Seven ratios (Table 1) were calculated based on laboratory tests and evaluated as classification factors for seven outcomes (Figure 1).

### 2.3. Statistical Analysis

**Figure 2:**
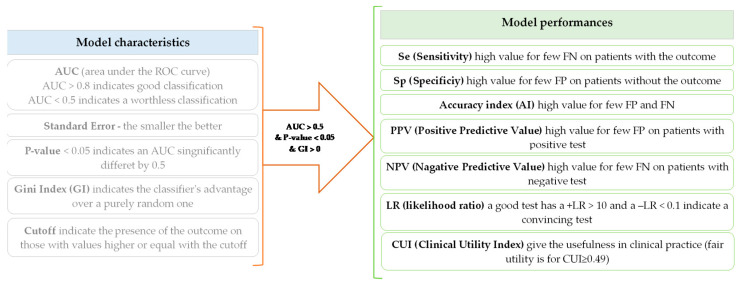
Model characteristics vs. model performances. FN = false negative; FP = false positive.

The receiver operating characteristic (ROC) analysis was performed in two steps: (1) model generation and (2) assessment of model performances (Figure 2). The model performance was checked only for those models with an area under the ROC curve (AUC) significantly higher than 0.5 and Gini index greater than 0. Seven indicators were used to evaluate the performances of the classification models. The values of the estimates were accompanied by the associated 95% confidence interval [24]. A classification model was considered clinically relevant whenever the Clinical Utility Index (CUI) proved greater than or equal to 0.49. A +CUI ≥ 0.49 indicates a test with abilities to accurately identify the outcome of interest. The greater the +CUI value, the better the power of ruling in (confirmation). A −CUI ≥ 0.49 indicates a test with abilities in screening; the greater the value, the better the power of ruling out (Figure 2).

The classification models using ROC analysis for NLR, dNLR and PLR were previously reported for the M stages [25]. However, we considered it worth investigating SII and PNI in correlation with the M stage, and these results are reported in the present study.

Statistical analysis was performed with IBM SPSS software version 26 (IBM SPSS, Armonk, NY, USA, trial version). The main characteristics of the sample were reported as mean (standard deviation) for normally distributed data, median (Q1 to Q3, where Q is the quartile) for quantitative data whenever they were proved not to follow the normal distribution and number (percent) for qualitative data. The Youden index (YI = Se + Sp − 1, where Se is sensitivity and Sp is specificity) was calculated to determine the optimal cutoff values. The corresponding binary variables were derived using the cutoff values for each investigated ratio, and the model performances were assessed for each outcome. The performance indicators were calculated using the following online resource: https://statpages.info/ctab2x2.html (accessed on 21 August 2020). A *p*-value less than 0.05 was considered to indicate an AUC statistically different by 0.5. Clinical utility for ruling in (confirmation) or ruling out (exclusion) was tested by combining classification using the OR operator based on individual cutoffs whenever the *p*-value was < 0.10.

Binomial logistic regression analysis was performed, considering the ROC thresholds for each outcome and constructing dichotomial secondary variables for each outcome of interest. All candidate ratios with AUCs significantly different by 0.5 in ROC analysis, age and gender were included in the multivariable logistic regression models. Significant predictors among evaluated ratios were identified using the Wald forward method in the first step of regression analysis. In the second step, age and gender were included as covariates to the identified predictors, and the enter method was applied to construct the models. The prediction ability for each investigated outcome was expressed as an odds ratio (OR) and associated 95% confidence intervals. The Hosmer and Lemeshow test was reported as a metric for the overall model performances, and a *p*-value less than 0.05 indicated a poor fit.

## 3. Results

In total, 1688 patients, 19 to 95 years old, with colorectal cancer were analyzed. The majority of the evaluated patients were males (978/1688, 57.9%). The death rate was equal to 3.1% (52/1688). The main characteristics of the evaluated cohort are presented in Table 2.

Two out of seven evaluated ratios proved borderline classification performances as prognostic factors for death, namely, NLR and dNLR (Table 3, *p* < 0.05). Both ratios had modest specificity (61.8% [59.4 to 64.2] for NLR, and 73.3% [71.1 to 75.4] for dNLR) but high negative predictive value (NLR: 98.0 [97.1 to 98.8] for NLR, and 97.8 [97.0 to 98.6] for dNLR), being fair as screening tools (−CUI of 0.605 [0.586 to 0.625] for NLR, and 0.717 [0.700 to 0.733] for dNLR). However, +LR is less than 2 and −LR is higher than 0.6, showing low performances as predictors. The AUC of LMR supports the existence of lower values for dead (median = 3.00, (Q1 to Q3) = 1.86 to 4.92, {min to max} = {0.30 to 12.76}) as compared to living subjects (median = 3.55, (Q1 to Q3) = 2.48 to 4.85, {min to max} = {0.28 to 15.21}), with a tendency to statistical significance (Mann–Whitney test: Z statistics = −1.34, *p*-value = 0.0819). Combining NLR, AGR and PNI led to a sensitivity of 90.4% [82.4 to 98.4]) and a specificity of 29.9% [37.7 to 32.1], but with poor utility both for case finding and screening (+CUI = 0.04 [0.000 to 0.084], −CUI = 0.30 [0.367 to 0.325]).

Despite the AUCs being significantly different by 0.5 for NLR, dNL and SII for the T stage classification (Table 4), the models’ performances do not support their clinical utility (Table 5).

The AUC for LMR is significantly lower than 0.5, indicating the presence of lower values for those with advanced T stage (T3 and T4, median = 3.40, (Q1 to Q3) = (2.43 to 4.60); {min to max} = {0.28 to 14.17}) as compared to those of early T stage (T1 and T2, median = 3.98, (Q1 to Q3) = (2.87 to 5.49); {min to max} = {0.56 to 15.21}). The observed differences between patients with advanced T stage proved statistically significant (Mann–Whitney test: Z statistics = −5.13, *p*-value < 0.0001). The classification combining NLR and SII for T stage does not lead to clinical utility improvement, the classification model being similar to individual ratios, namely, fair for case finding (+CUI = 0.50 [0.468 to 0.533]).

Only the SII ratio showed AUCs significantly higher than 0.5 (Table 6) for the presence of metastasis, with fair utility for screening (Table 7). Fair utility for screening was also obtained for NLR (+CUI = 0.20 [0.144 to 0.250] and –CUI = 0.58 [0.561 to 0.604]), dNLR (+CUI = 0.20 [0.143 to 0.247] and –CUI = 0.56 [0.532 to 0.577]) and PLR (+CUI = 0.11 [0.057 to 0.171] and –CUI = 0.63 [0.614 to 0.652]). The AUCs of LMR and PNI indicate the existence of significantly lower values among those with metastasis, as compared to those without metastasis. The statistical summary for LMR according to the M stage was as follows: median = 3.18, (Q1 to Q3) = (1.78 to 4.51); {min to max} = {0.28 to 14.00} for those with metastasis, and median = 3.67, (Q1 to Q3) = (2.65 to 4.94); {min to max} = {0.39 to 15.21} for those without metastatic disease, the difference being statistically significant (Mann–Whitney test: Z statistics = −5.77, *p*-value < 0.0001). The statistical summary for PNI according to the M stage was as follows: median = 31.01, (Q1 to Q3) = (28.01 to 35.00); {min to max} = {18.01 to 47.01} for those with metastasis, and median = 32.01, (Q1 to Q3) = (28.01 to 36.00); {min to max} = {18.01 to 64.01} for those without metastasis, the difference being statistically significant (Mann–Whitney test: Z statistics = −2.36, *p*-value = 0.0185).

The classification model obtained by combining NLR with SII shows poor abilities for case finding (+CUI = 0.21 [0.106 to 0.261]) and fair capabilities for screening (−CUI = 0.52 [0.501 to 0.547]).

NLR, PLR and SII proved performant in ROC analysis for the evaluation of the chemo- or radiotherapy impact on patients with colorectal cancer (Table 8), but only PLR demonstrated fair clinical utility (Table 9). The classification model obtained by combining the ratios mentioned earlier shows a model with very poor capabilities both for case finding (+CUI = 0.13 [0.088 to 0.180]) and screening (−CUI = 0.33 [0.297 to 0.355]).

The AUC of LMR indicates significantly lower values among those with chemo- or radiotherapy compared to those without. The statistical summary for LMR according to chemo- or radiotherapy was as follows: median = 3.00, (Q1 to Q3) = (2.19 to 4.11); {min to max} = {0.56 to 11.90} for those with, and median = 3.65, (Q1 to Q3) = (2.53 to 4.98); {min to max} = {0.28 to 15.21} for those without, the difference being statistically significant (Mann–Whitney test: Z statistics = −5.16, *p*-value < 0.0001).

All of the investigated ratios proved low abilities as predictors for postoperative complications (Table S1, Supplementary Material), feeding resumption (Table S2, Supplementary Material) or transit resumption (Table S3, Supplementary Material).

The multivariable regression models showed significant prediction value for four out of seven investigated outcomes (Table 10). The dNLR proved to be a predictor of death, independent of gender. NLR and LMR exhibit predictive potential for the T stage, independent of age or gender, while PNI displays predictive potential for the M stage.

## 4. Discussion

The evaluation of ratios derived from the routinely performed serum markers shows limited performances and clinical utility, when individually used as prognostic factors for patients with colorectal cancer. Some of the investigated ratios proved fair utility for screening (NLR and dNLR for death and T stage, LMR and SII for T stage and M stage, PNI for M stage), but the associated performance metrics do not support their utility as individual predictors. The ROC analysis allowed us to identify relevant cutoffs for the investigated ratios, which were used to construct secondary variables and predictors in multivariable regression analysis. Thus, dNLR proved to be a significant predictor for death, independent of gender and dependent on age, and NLR and LMR proved to be predictors for the T stage, independent of age or gender, while NLR and PNI showed abilities as predictors for the M stage, independent of age, but dependent on gender.

As expected, the predominant number of cases of colorectal cancer in our study was found in males (Table 2). Studies indicate similar results on the incidence of colorectal cancer. Siegel et al. analyzed SEER (Surveillance, Epidemiology and End Results) over five years and reported an incidence of 40.7 per 100,000 people for colorectal cancer with an increased male predisposition [26]. The death rate during hospitalization, in our study, was 3.1%, being in the trend with the data reported worldwide (4.3 per 100,000 people) [27]. In developed and developing countries, the death rate from colorectal cancer has begun to decline since 1980. At least from the diagnosis point of view, this may be explained by the screening methods that suffered tremendous improvements. Detecting colonic polyps endoscopically and the possibility of minimally invasive resections have led to early cancer identification [28]. Secondly, from the treatment point of view, new neoadjuvant therapy methods emerged in the last decade that have increased these patients’ survival. It is known that age is an important predictor of colorectal cancer. The incidence is high in the fourth–sixth decades of life, and age-specific incidence rates increase in each subsequent decade thereafter [29]. The mean age in our cohort of patients was 65 (Table 2), which fits the literature trends. Most of the evaluated subjects were stage T3, followed by stages T2 and T4 at the time of diagnosis and most patients were stage M0 according to the TNM (T—size or direct extent of the primary tumor, N—degree of spread to regional lymph nodes, M—presence of distant metastasis) classification (Table 2). These results reveal that people still present in health care institutions in an advanced stage of the disease, resulting in a reserved long-term prognosis [30].

Approximately 50% of the patients in our study developed mild postoperative complications, namely, hemorrhage and surgical infections (Table 2), which were solved during hospitalization. Complications can lead to an increased hospital stay, morbidity and mortality. Pre- and postoperative risk factors may predict the incidence of these complications [31]. In our cohort, the diet resumption was, in the majority of cases (97%), in the first three days postoperatively (Table 2), followed by intestinal transit resumption in the first four days (Table 2), considered expected, normal evolution, according to the general data in the literature [32]. Analyzing the combined statistical significance between NLR, AGR and PNI led to a low case finding and screening sensitivity, similar to other researchers’ findings [33].

The study highlights that either of the evaluated ratios proved to be statistically associated with certain outcomes considered (Table 3, Table 4, Table 5, Table 6, Table 7, Table 8, Table 9 and Table 10). Moreover, it has been shown that some of the ratios have good prognostic potential for specific outcomes, considering patients with colorectal cancer (Table 10). NLR and dNLR can be used as predictors of death (Table 3). Ozdemir et al. showed that elevated preoperative NLR and dNLR values are associated with lower survival in colorectal cancer patients receiving surgical interventions and may play an important role in choosing the appropriate treatment [34]. LMR proved lower values in patients who died than those who survived in our cohort. LMR had previously been reported as a mild predictive factor in colorectal cancer, with low LMR values associated with death [33,35]. More studies are needed in order to correctly identify whether LMR may be used as a prognostic factor in colorectal cancer.

We demonstrated that the association between NLR, dNLR and the T stage is statistically significant with potential clinical utility. Further, in the case of LMR, it has been shown that its values are inversely proportional to the T stage, with good statistical significance (Table 4). The clinical utility of the combination between NLR and SII regarding the T stage proves similar values with these ratios individually evaluated [36].

Analyzing the ratios based on metastasis’s presence, the SII proved some performances as an individual predictor. Moreover, NLR, dNLR and PLR have proven useful for the screening of subjects with metastasis. PNI and LMR showed lower values in patients with metastasis than those without and proved to be statistically significant (Table 6). Higher NLR and dNLR values were reported in patients with advanced disease than in the early stages [37,38]. Özgehan et al. reported that NLR showed significantly high values in patients with positive lymph nodes [39]. LMR has been proposed as a prognostic factor for metastasis in colorectal cancer but did not prove such clinical reliability as NLR or dNLR [40]. Lu et al. [41] stated that TNM in association with PLR has better prognostic capacity than the TNM classification alone and that PLR can be used as an additional parameter to TNM staging. As for PNI, no studies have been identified in the literature to compare this ratio and analyze it in association with metastasis in colorectal cancer.

NLR, PLR and SII were demonstrated as individual predictors for neoadjuvant therapy, both chemotherapy and radiation or concomitant radio-chemotherapy (Table 8). PLR demonstrated fair clinical utility with higher sensitivity and specificity than the other ratios evaluated (Table 9). Lino-Silva et al. [42] reported limited utility of NLR as a prognostic marker in patients with locally advanced rectal cancer, who received neoadjuvant chemoradiotherapy. Chua et al. highlighted NLR as a potentially useful clinical biomarker for patients undergoing chemotherapy or radiation, both as neoadjuvant or adjuvant systemic treatment [43]. Elevated NLR and PLR may have a close association with low survival in colorectal cancer and thus may be used as prognostic indicators for early identification of colorectal cancer patients with poor prognosis [44]. Lin et al. reported that an increased value of LMR before systemic treatment is a favorable prognostic factor for these patients’ survival. Changes in this parameter before and after chemotherapy show the benefit of neoadjuvant treatment [45]. The classification model obtained by combining NLR, PLR and SII regarding the impact of neaoadjuvant chemoradiotherapy does not elevate the models’ statistical value, proving equal benefit with the independenly selected ratios [46].

The evaluated ratios demonstrated low or mild capacities as predictors of postoperative complications. High values of NLR, dNLR and PLR, or, on the other hand, low values of LMR, AGR and PNI may alert the clinicians to possible complications that may occur postoperatively, but with poor sensitivity and specificity. Four of the investigated outcomes proved to be significantly associated with at least one ratio, dependent or not on age and gender (Table 10). Therefore, dNLR turned out to be a strong predictor of survival, dependent on age and independent of gender. Thus, a patient with dNLR ≥ 3.125 had almost twice the odds of dying, while the increase in age with one year increased the chances of death by 4%. NLR and LMR are significantly associated with the tumor’s local stage, independent of gender and age. A pre-treatment value of NLR higher than 3.105 increases the odds to be in a more advanced T stage, while an increase wof 1 in the LMR ratio decreases this chance. An NLR value higher than 4.255 increases by almost three times the odds of metastasis, while an increase of one unit in PNI decreases the odds of metastasis. Several studies have highlighted the importance of these biological markers in the prognosis of colorectal cancer and are used in postoperative monitorization of this pathology [47,48,49,50,51]. The results reported in Table 10 could be used to predict a specific outcome if the values associated with the independent variable are known, but the performances in the prediction need to be validated on external samples and considering the influence of all possible confounding factors.

### Study Limitations and Further Research

The results of our study must be interpreted with caution and a number of limitations should be borne in mind. First, the accuracy of the data from the medical charts could not be verified while the missing data could not be controlled; therefore, a selection bias could be noted. Secondly, by retrospective collection of data, no control for confounding factors was possible and thus the identified associations could be misleading, or over- or under-estimated. As a measure to reduce confoundings, the regression analysis was conducted to investigate the effect of gender and age, but unfortunately other confounders that could influence the values of the investigated ratios (such as smoking, inflammatory diseases, drugs, genetic, diabetes, advanced age) remained unknown [52]. Thirdly, misclassification of some investigated outcomes, such as T stage and M stage, could also be acknowledged, since different imaging methods were used to evaluate the patients and different physicians interpreted the imaging examinations. It could be expected that the identified fair clinical utility will be slightly greater in reality. The evaluation of the proposed inflammatory and associated ratios in the context of prospective inclusion of patients in the study, with a standardized evaluation protocol (classification of outcomes), and measurement or collection of all possible confounders could better reflect the reality regarding the clinical utility of these ratios. Furthermore, dynamic evaluation of ratios (e.g., before and after surgery, before and after neoadjuvant therapy) should be conducted to reveal their clinical relevance and we are currently taking it into consideration.

## 5. Conclusions

The evaluated ratios possess individual prognostic value at specific cutoffs for certain outcomes considered, showing fair utility as screening markers, and could play a substantial role in the therapeutical management of patients, but further studies to prove their clinical relevance are needed. Our study highlighted that dNLR is a reliable marker for the prognosis of survival for colorectal cancer patients who have undergone surgical treatment. NLR, LMR and PNI are accurate prognostic markers, significantly influenced by the disease’s T and M stages. PLR provides important information about the effect of neoadjuvant treatment on patients with colorectal cancer. Our results support the rationale for conducting prospective multicentric studies to demonstrate the ratios’ clinical value and to transfer the findings into the clinical management of patients with colorectal cancer.

## Figures and Tables

**Table 1:** Ratios evaluated as prognostic factors in patients with colorectal cancer.

Ratio	Abb	Formula	Cutoff Values
Neutrophil-to-Lymphocyte Ratio	NLR	(Absolute Neutrophil Count)/(Absolute Lymphocyte Count)	<2.5
Derived Neutrophil-to-Lymphocyte Ratio	dNLR	(Absolute Neutrophil Count)/[(Absolute Leucocyte Count) − (Absolute Neutrophil Count)]	<2
Platelet-to-Lymphocyte Ratio	PLR	(Absolute Platelet Count)/(Absolute Lymphocyte Count)	<150
Lymphocyte-to-Monocyte Ratio	LMR	(Absolute Lymphocyte Count)/(Absolute Monocyte Count)	<2.5
Albumin-to-Globulin Ratio	AGR	Albumin/[(Total Proteins) − Albumin]	>1.5
Systemic Immune Inflammation Index	SII	(Absolute Neutrophil Count) × (Absolute Platelet Count)/(Absolute Lymphocyte Count)	390 × 10^9^ cells/L
Prognostic Nutritional Index	PNI	10 × Serum Albumin (g/dL) + 0.005 × Lymphocyte Count (per mm^3^) [23]	n.a.

n.a. = not available.

**Table 2:** Demographics, stage of the disease and perioperative characteristics of the patients.

Characteristics	Value
Age, years ^a^	65.1 (10.6)
Living area ^b^	
Urban	1180 (69.9)
Rural	508 (30.1)
T stage ^b^	
T1	74 (4.4)
T2	376 (22.3)
T3	763 (45.2)
T4	473 (28)
TIS	2 (0.1)
M stage ^b^	
M0	1270 (75.2)
M1	418 (24.8)
Complications, yes ^b^	947 (56.1)
Chemo- or radiotherapy ^b^	257 (15.2)
Feeding resumption	
Median (Q1 to Q3)	2 (1 to 2)
In the first 3 days postoperative	1631 (96.6)
Transit resumption	
Median (Q1 to Q3)	2 (2 to 3)
In the first 4 days postoperative ^b^	1632 (96.7)

^a^ mean (standard deviation); ^b^ no. (%); Q1 = the first quartile; Q3 = the third quartile.

**Table 3:** Performance of the area under the ROC curve for death.

Ratio	AUC [95% CI]	StdErr	*p*-Value	Cutoff	GI
NLR	0.593 [0.503 to 0.683]	0.046	0.0423	4.005	0.186
dNLR	0.592 [0.501 to 0.683]	0.047	0.0476	3.125	0.184
PLR	0.540 [0.446 to 0.635]	0.048	0.4027	213.510	0.081
LMR	0.429 [0.337 to 0.522]	0.047	0.1334	5.465	−0.142
AGR	0.571 [0.498 to 0.644]	0.037	0.0578	0.815	0.142
SII	0.566 [0.467 to 0.664]	0.050	0.1913	1447.044	0.132
PNI	0.425 [0.339 to 0.510]	0.044	0.0836	37.004	−0.151

AUC = area under the ROC curve; 95% CI = 95% lower to upper bound; StdErr = standard error; GI = Gini index; NLR = neutrophil-to-lymphocyte ratio; dNLR = derived neutrophil-to-lymphocyte ratio; PLR = platelet-to-lymphocyte ratio; LMR = lymphocyte-to-monocyte ratio; AGR = albumin-to-globulin ratio; SII = Systemic Immune Inflammation Index; PNI = Prognostic Nutritional Index.

**Table 4:** Performance of the area under the ROC curve for T stage.

Ratio	AUC [95% CI]	StdErr	*p*-Value	Cutoff	GI
NLR	0.578 [0.547 to 0.609]	0.01593	<0.0001	3.105	0.156
dNLR	0.562 [0.531 to 0.593]	0.01592	0.0001	2.155	0.125
PLR	0.531 [0.500 to 0.562]	0.01594	0.0525	123.865	0.062
LMR	0.418 [0.387 to 0.449]	0.01573	<0.0001	16.210	−0.163
AGR	0.494 [0.463 to 0.526]	0.01624	0.7276	0.505	−0.011
SII	0.555 [0.524 to 0.587]	0.01600	0.0006	764.958	0.110
PNI	0.499 [0.467 to 0.53]	0.01628	0.9283	28.509	−0.003

AUC = area under the ROC curve; 95% CI = 95% lower to upper bound; StdErr = standard error; GI = Gini index; NLR = neutrophil-to-lymphocyte ratio; dNLR = derived neutrophil-to-lymphocyte ratio; PLR = platelet-to-lymphocyte ratio; LMR = lymphocyte-to-monocyte ratio; AGR = albumin-to-globulin ratio; SII = Systemic Immune Inflammation Index; PNI = Prognostic Nutritional Index.

**Table 5:** T stage: performance metrics as support for the classification model.

Metrics	NLR	dNLR	SII
Se	60.6 [59.1 to 62]	61.7 [60.2 to 63.1]	54.4 [53 to 55.9]
Sp	53.1 [49.1 to 57.1]	50.2 [46.2 to 54.3]	54.5 [50.4 to 58.5]
AI	41.4 [39.3 to 43.6]	41.4 [39.2 to 43.5]	45.6 [43.4 to 47.7]
PPV	78.1 [76.3 to 80]	77.4 [75.6 to 79.3]	76.8 [74.7 to 78.8]
NPV	32.7 [30.2 to 35.2]	32.1 [29.6 to 34.7]	30.2 [27.9 to 32.4]
+LR	1.3 [1.2 to 1.4]	1.2 [1.1 to 1.4]	1.2 [1.1 to 1.3]
−LR	0.7 [0.7 to 0.8]	0.8 [0.7 to 0.9]	0.8 [0.8 to 0.9]
+CUI	0.473 [0.440 to 0.507]	0.477 [0.444 to 0.510]	0.418 [0.383 to 0.453]
−CUI	0.174 [0.141 to 0.207]	0.160 [0.127 to 0.193]	0.164 [0.133 to 0.196]

NLR = neutrophil-to-lymphocyte ratio; dNLR = derived neutrophil-to-lymphocyte ratio; SII = Systemic Immune Inflammation Index; Se = sensitivity; Sp = specificity; AI = accuracy index; PPV = positive predictive value; NPV = negative predictive value; +LR = positive likelihood ratio; −LR = negative likelihood ratio; +CUI = positive Clinical Utility Index; −CUI = negative Clinical Utility Index.

**Table 6:** Performance of the area under the ROC curve for the M stage.

Ratio	AUC [95% CI]	StdErr	*p*-Value	Cutoff	GI
LMR	0.406 [0.373 to 0.440]	0.017	<0.0001	12.990	−0.188
AGR	0.487 [0.455 to 0.518]	0.016	0.4003	0.855	−0.027
SII	0.618 [0.586 to 0.649]	0.016	<0.0001	1037.416	0.235
PNI	0.462 [0.431 to 0.493]	0.016	0.0157	65.010	−0.077

AUC = area under the ROC curve; 95% CI = 95% lower to upper bound; StdErr = standard error; GI = Gini index; LMR = lymphocyte-to-monocyte ratio; AGR = albumin-to-globulin ratio; SII = Systemic Immune Inflammation Index; PNI = Prognostic Nutritional Index.

**Table 7:** M stage: performance metrics as support for the classification model.

Metrics	SII
Se	51.7 [47.5 to 55.8]
Sp	67.8 [66.4 to 69.2]
AI	36.2 [34.1 to 38.3]
PPV	34.6 [31.7 to 37.3]
NPV	81 [79.3 to 82.6]
+LR	1.6 [1.4 to 1.8]
−LR	0.7 [0.6 to 0.8]
+CUI	0.18 [0.127 to 0.231]
−CUI	0.55 [0.527 to 0.571]

SII = Systemic Immune Inflammation Index; Se = sensitivity; Sp = specificity; AI = accuracy index; PPV = positive predictive value; NPV = negative predictive value; +LR = positive likelihood ratio; −LR = negative likelihood ratio; +CUI = positive Clinical Utility Index; −CUI = negative Clinical Utility Index.

**Table 8:** Performance of the area under the ROC curve for chemo- or radiotherapy.

Ratio	AUC [95% CI]	StdErr	*p*-Value	Cutoff	GI
NLR	0.572 [0.535 to 0.609]	0.019	0.0001	3.135	0.144
dNLR	0.523 [0.486 to 0.560]	0.019	0.2216	1.975	0.046
PLR	0.615 [0.576 to 0.655]	0.020	<0.0001	198.320	0.231
LMR	0.399 [0.363 to 0.435]	0.018	<0.0001	0.555	−0.202
AGR	0.470 [0.432 to 0.508]	0.019	0.1187	2.100	−0.061
SII	0.554 [0.516 to 0.592]	0.019	0.0055	736.775	0.107
PNI	0.519 [0.48 to 0.557]	0.020	0.3487	33.014	0.037

AUC = area under the ROC curve; 95% CI = 95% lower to upper bound; StdErr = standard error; GI = Gini index; NLR = neutrophil-to-lymphocyte ratio; dNLR = derived neutrophil-to-lymphocyte ratio; PLR = platelet-to-lymphocyte ratio; LMR = lymphocyte-to-monocyte ratio; AGR = albumin-to-globulin ratio; SII = Systemic Immune Inflammation Index; PNI = Prognostic Nutritional Index.

**Table 9:** Chemo- or radiotherapy: performance metrics as support for the classification model.

Metrics	NLR	PLR	SII
Se	67.7 [62 to 73]	52.1 [46.4 to 57.8]	64.6 [58.8 to 70]
Sp	45.8 [44.8 to 46.8]	67.8 [66.8 to 68.8]	48.2 [47.2 to 49.2]
AI	50.8 [49.2 to 52.6]	34.6 [32.9 to 36.4]	49.3 [47.6 to 51]
PPV	18.3 [16.8 to 19.8]	22.5 [20 to 25]	18.3 [16.7 to 19.8]
NPV	88.8 [86.8 to 90.6]	88.7 [87.4 to 90.1]	88.3 [86.5 to 90.1]
+LR	1.3 [1.1 to 1.4]	1.6 [1.4 to 1.9]	1.2 [1.1 to 1.4]
−LR	0.7 [0.6 to 0.8]	0.7 [0.6 to 0.8]	0.7 [0.6 to 0.9]
+CUI	0.124 [0.076 to 0.173]	0.117 [0.061 to 0.174]	0.118 [0.069 to 0.167]
−CUI	0.407 [0.381 to 0.433]	0.602 [0.581 to 0.622]	0.426 [0.400 to 0.452]

NLR = neutrophil-to-lymphocyte ratio; PLR = platelet-to-lymphocyte ratio; SII = Systemic Immune Inflammation Index; Se = sensitivity; Sp = specificity; AI = accuracy index; PPV = positive predictive value; NPV = negative predictive value; +LR = positive likelihood ratio; −LR = negative likelihood ratio; +CUI = positive Clinical Utility Index; −CUI = negative Clinical Utility Index.

**Table 10:** Predictors by multivariable logistic regression analysis for different outcomes of patients with colorectal cancer.

Predictor	β	SEβ	Wald’s χ^2^	*p*-Value	OR (95% CI)	Hosmer and Lemeshow
***Death***						4.85 (0.7740)
Age, years	0.043	0.015	8.84	0.0030	1.044 (1.015 to 1.074)	
dNLR ≥ 3.125	0.909	0.284	10.21	0.0014	2.481 (1.421 to 4.331)	
Gender, male	0.087	0.290	0.091	0.7626	1.091 (0.619 to 1.925)	
Constant	−6.68	1.020	43.86	<0.0001		
***T stage***						6.48 (0.5940)
Age, years	−0.010	0.005	3.67	0.0553	0.990 (0.979 to 1.000)	
Gender, male	0.097	0.113	0.75	0.3880	1.102 (0.884 to 1.374)	
NLR ≥ 3.105	0.328	0.137	5.73	0.0167	1.389 (1.061 to 1.817)	
LMR	−0.084	0.030	7.88	0.0050	0.919 (0.867 to 0.975)	
Constant	1.802	0.411	19.22	<0.0001		
***M stage***						8.19 (0.4151)
Age, years	−0.007	0.005	1.60	0.2054	0.993 (0.983 to 1.004)	
Gender, male	0.308	0.120	6.59	0.0102	1.360 (1.076 to 1.720)	
NLR ≥ 4.255	0.984	0.117	71.27	<0.0001	2.674 (2.128 to 3.360)	
PNI	−0.030	0.012	6.38	0.0115	0.970 (0.947 to 0.993)	
Constant	−0.285	0.532	0.29	0.593		
***Chemo- or radiotherapy***						4.52 (0.8076)
Age, years	−0.036	0.006	31.98	<0.0001	0.965 (0.953 to 0.977)	
Gender, male	0.228	0.144	2.50	0.1138	1.256 (0.947 to 1.665)	
PLR ≥ 198.32	0.675	0.149	20.58	<0.0001	1.964 (1.467 to 2.630)	
LMR	−0.111	0.039	8.00	0.0047	0.895 (0.829 to 0.967)	
Constant	0.541	0.455	1.41	0.2351		

β = coefficient; SEβ = standard error of the coefficient; OR = odds ratio; (95% CI) = 95% confidence intervals (lower bound to upper bound); dNLR = derived neutrophil-to-lymphocyte ratio; NLR = neutrophil-to-lymphocyte ratio; LMR = lymphocyte-to-monocyte ratio; PNI = Prognostic Nutritional Index; PLR = platelet-to-lymphocyte ratio.

## Data Availability

The data presented in this study are available on request from the corresponding authors. The data are not publicly available due to privacy restrictions.

## Supplementary Materials

The following are available online at https://www.mdpi.com/2075-4418/11/3/566/s1, Table S1: Performance of the area under the ROC curve for complications, Table S2: Performance of the area under the ROC curve for feeding resumption, Table S3: Performance of the area under the ROC curve for transit resumption.
