# Supplementary material for: Abilities of Pre-Treatment Inflammation Ratios as Classification or Prediction Models for Patients with Colorectal Cancer
Source: Diagnostics (Basel). 2021 Mar 21;11(3):566. doi: 10.3390/diagnostics11030566 (PMC8003848; doi:10.3390/diagnostics11030566)
Supplement: Supplementary file 1 [file diagnostics-11-00566-s001.pdf]

Article

Prediction abilities of pre-treatment inflammation ratios as classification or prediction models for patients with colorectal cancer?

Andra Ciocan, Răzvan A. Ciocan, Nadim Al Hajjar, Claudia D. Gherman and Sorana D. Bolboacă

Table S1. Performance of the area under the ROC curve for complications.

Ratio	AUC [95% CI]	StErr	p-value	Cutoff	GI
NLR	0.506 [0.478 to 0.534]	0.014	0.6731	2.015	0.012
dNLR	0.520 [0.492 to 0.548]	0.014	0.1585	2.085	0.040
PLR	0.496 [0.468 to 0.524]	0.014	0.7891	335.460	-0.008
LMR	0.509 [0.481 to 0.537]	0.014	0.5261	4.835	0.018
AGR	0.485 [0.457 to 0.512]	0.014	0.2775	0.985	-0.031
SII	0.501 [0.473 to 0.528]	0.014	0.9662	420.944	0.001
PNI	0.495 [0.468 to 0.523]	0.014	0.7366	33.011	-0.009

AUC = area under the ROC curve; 95%CI = 95% lower to upper bound; StdErr = standard error; GI = Gini index; NLR = Neutrophil-to-Lymphocyte Ratio; dNLR = derived Neutrophil-to-Lymphocyte Ratio; PLR = Platelet-to-Lymphocyte Ratio; LMR = Lymphocyte-to-Monocyte Ratio; AGR= Albumin-to-Globulin Ratio; SII= Systemic Immune Inflammation Index; PNI = Prognostic Nutritional Index

Table S2. Performance of the area under the ROC curve for feeding resumption.

Ratio	AUC [95% CI]	StErr	p-value	Cutoff	GI
NLR	0.511 [0.435 to 0.588]	0.039	0.7688	4.925	0.023
dNLR	0.515 [0.435 to 0.595]	0.041	0.7129	3.145	0.030
PLR	0.491 [0.419 to 0.563]	0.037	0.8033	93.755	-0.018
LMR	0.576 [0.487 to 0.665]	0.045	0.0950	5.625	0.152
AGR	0.545 [0.471 to 0.62]	0.038	0.2330	0.945	0.091
SII	0.5 [0.419 to 0.581]	0.041	0.9978	1448.234	0.000
PNI	0.49 [0.415 to 0.565]	0.038	0.7927	31.006	-0.020

AUC = area under the ROC curve; 95%CI = 95% lower to upper bound; StdErr = standard error; GI = Gini index; NLR = Neutrophil-to-Lymphocyte Ratio; dNLR = derived Neutrophil-to-Lymphocyte Ratio; PLR = Platelet-to-Lymphocyte Ratio; LMR = Lymphocyte-to-Monocyte Ratio; AGR= Albumin-to-Globulin Ratio; SII= Systemic Immune Inflammation Index; PNI = Prognostic Nutritional Index

Table S3. Performance of the area under the ROC curve for transit resumption.

Ratio	AUC [95% CI]	StdErr	p-value	Cutoff	GI
NLR	0.517 [0.44 to 0.593]	0.039	0.6683	3.075	0.054
dNLR	0.527 [0.451 to 0.603]	0.039	0.4879	149.940	0.036
PLR	0.518 [0.442 to 0.594]	0.039	0.6451	6.875	-0.005
LMR	0.497 [0.415 to 0.579]	0.042	0.9477	0.815	-0.033
AGR	0.483 [0.406 to 0.56]	0.039	0.6713	1770.213	0.021
SII	0.51 [0.431 to 0.589]	0.040	0.7991	36.006	0.001
PNI	0.501 [0.414 to 0.588]	0.044	0.9877	0.000	0.000

AUC = area under the ROC curve; 95%CI = 95% lower to upper bound; StdErr = standard error; GI = Gini index; NLR = Neutrophil-to-Lymphocyte Ratio; dNLR = derived Neutrophil-to-Lymphocyte Ratio; PLR = Platelet-to-Lymphocyte Ratio; LMR = Lymphocyte-to-Monocyte Ratio; AGR= Albumin-to-Globulin Ratio; SII= Systemic Immune Inflammation Index; PNI = Prognostic Nutritional Index
